# Dietary adherence to the Mediterranean diet pattern in a randomized clinical trial of patients with quiescent ulcerative colitis

**DOI:** 10.3389/fnut.2022.1080156

**Published:** 2022-12-21

**Authors:** Natasha Haskey, Rachel C. K. Shim, Alexander Davidson-Hunt, Jiayu Ye, Sunny Singh, Levinus A. Dieleman, Kevan Jacobson, Sanjoy Ghosh, Deanna L. Gibson

**Affiliations:** ^1^Department of Biology, University of British Columbia - Okanagan Campus, Kelowna, BC, Canada; ^2^Department of Public Health Sciences, The Dalla Lana School of Public Health, University of Toronto, Toronto, ON, Canada; ^3^Diabetes Center, University of California, San Francisco, San Francisco, CA, United States; ^4^Division of Gastroenterology, Department of Medicine, University of British Columbia, Vancouver, BC, Canada; ^5^Division of Gastroenterology, Department of Medicine, University of Alberta, Edmonton, AB, Canada; ^6^Division of Gastroenterology, Department of Pediatrics, Hepatology and Nutrition and British Columbia Children’s Hospital Research Institute, University of British Columbia, Vancouver, BC, Canada; ^7^Faculty of Medicine, University of British Columbia - Okanagan Campus, Kelowna, BC, Canada

**Keywords:** diet, compliance, Mediterranean diet, ulcerative colitis, Healthy Eating Index-2015, Mediterranean diet serving score

## Abstract

**Background:**

The Mediterranean diet pattern (MDP) is believed to improve health and promote balanced inflammation and metabolism. While unknown, compelling evidence suggests that MDP could benefit patients with inflammatory bowel disease (IBD). We aimed to evaluate the level of diet adherence, diet quality, and nutritional adequacy of the MDP in patients with Ulcerative Colitis (UC).

**Methods:**

Adult participants (*n* = 32) with quiescent UC were randomized to follow a MDP (*n* = 18) or Canadian Habitual Diet (CHD) (*n* = 14) for 12 weeks. The MDP participants received tailored nutrition education from a Registered Dietitian. Demographic, clinical data, medical history, and quality of life were assessed with the Short Inflammatory Bowel Disease Questionnaire (SIBDQ), dietary adherence with the Mediterranean Diet Serving Score (MDSS), diet quality via the Healthy Eating Index-2015 (HEI-2015), and dietary intake (ASA-24) were completed at baseline and week 12.

**Results:**

Participants’ diets were analyzed (MDP *n* = 15, CHD *n* = 13). The MDP (*n* = 10, 67%) achieved a high level of adherence (MDSS score between 16 and 24) vs. CHD (*n* = 3), (*p* = 0.030). HEI-2015 significantly increased from baseline to week 12 (*p* = 0.007) in the MDP and was significantly higher at week 12 compared to the CHD (*p* = 0.0001). The SIBDQ (bowel domain) showed reductions in the passage of large amounts of gas (*p* = 0.01) and improvements in tenesmus (*p* = 0.03) in the MDP. Despite enhanced diet quality and adherence in the MDP, females had inadequate intakes of calcium, iron, vitamin D, vitamin E, and choline and males had inadequate intakes of fiber, vitamin D, vitamin E, and choline. No adverse events were reported.

**Conclusion:**

With nutrition education, high adherence to the MDP was achieved without an increase in bowel symptoms. Following the MDP led to a higher diet quality; however, nutritional inadequacies were identified. Tailored dietary education focusing on nutrients of concern when following the MDP is recommended to ensure nutritional adequacy.

**Clinical trial registration:**

[www.ClinicalTrials.gov], identifier [NCT03053713].

## Introduction

Ulcerative colitis (UC), a form of inflammatory bowel disease (IBD), is a chronic inflammatory condition that produces contiguous mucosal inflammation of the colon (1). UC is characterized by ongoing and debilitating symptoms, including bloody diarrhea, abdominal pain, cramping, tenesmus, weight loss and fatigue (2). Finite medical therapeutic options combined with clinical remission being reached in less than half of patients leads to severe impacts on quality of life (3). Lifestyle modifications, such as diet, are of interest to patients. Reports indicate that up to 77% of patients restrict foods such as vegetables, fruits, legumes, whole grains, nuts, seeds, and dairy (4, 5). A dietary intervention that could improve inflammatory biomarkers and maintain clinical remission has the potential to prevent increases in dosage of medication, as well as avoid having to switch to biologics.

The Mediterranean Diet Pattern (MDP) is a potential therapeutic for IBD due to its ability to modulate the gut microbiome and inflammation (6–9). The MDP is rich in plant foods (cereals, fruits, vegetables, legumes, tree nuts, seeds, and olives), with olive oil as the principal source of added fat, high to moderate intakes of fish and seafood, moderate consumption of eggs, poultry, and dairy products (cheese and yogurt), low consumption of red meat and a moderate intake of alcohol (mainly red wine during meals) (10). The protective effect of the MDP is believed to come from the fatty acid profile [35% total fat: high in monounsaturated fatty acids (MUFA)] (mainly from olive oil), moderate saturated fatty acids (SFA), and low n-6 polyunsaturated fatty acids (PUFA and some n-3 PUFA), as well as polyphenols and fiber (11). Increased adherence to a MDP is necessary to realize the health benefits (12–14). Despite the potential benefits of the MDP in UC, the guidelines may be challenging for patients to follow due to self-imposed dietary restrictions.

There is a lack of prospective intervention trials evaluating diet adherence to the MDP in IBD. The clinical trials completed to date focus on retrospective, habitual dietary intake assessment, so the ability of a participant to follow the MDP with nutrition coaching by a dietitian is worth exploring. Considering the potential for the MDP to play a role in IBD management, this study aimed to evaluate dietary adherence to the MDP, the adequacy of nutrient intake, quality of the dietary pattern, and quality of life in patients with UC.

## Materials and methods

### Study design

**FIGURE 1:**
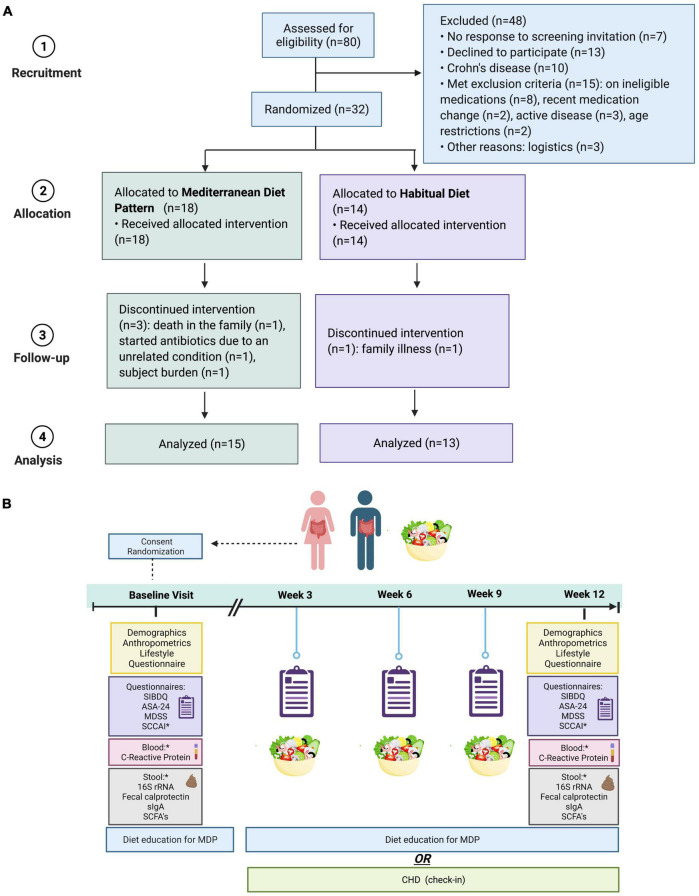
**(A)** Flow chart of recruitment. **(B)** Summary of the 12-week intervention of the MDP vs. CHD in patients with quiescent UC. SIBDQ, short inflammatory bowel disease questionnaire (quality of life); ASA-24, 24-h dietary recall; MDSS, Mediterranean diet serving score (compliance); SCCAI, Simple Clinical Colitis Activity Index (disease activity/symptoms); SCFAs, short-chain fatty acids; MDP, Mediterranean diet pattern (intervention); CHD, Canadian habitual diet (control); sIGA, secretory immunoglobulin A. *A component of the larger analysis of the same trial. Graphic prepared with Biorender.com.

This study is a secondary analysis of data derived from a randomized controlled trial registered with Clinical Trials NCT03053713. Participants provided demographic, anthropometric, survey, and dietary data for the original study to examine associations with the gut microbiome and inflammation (Figures 1A, B). For this secondary analysis, adherence to the MDP was the outcome of interest.

Adult participants (18–65 years) diagnosed with mild-moderate UC in remission (partial Mayo score 0–2) were recruited through outpatient gastroenterology clinics in British Columbia (the Okanagan Valley, Vancouver, and Victoria) and Edmonton, Alberta, Canada, from April 2017 to September 2021. The diagnosis of IBD was established according to the guidelines set by the British Society of Gastroenterology (15). To be enrolled in the study, participants prescribed oral 5-aminosalicylates, thiopurines, or methotrexate could not have dosage adjustments 2 months before starting the study. Participants taking biologics (infliximab, adalimumab) were accepted if there were no dosage adjustments 3 months before the study began. Exclusion criteria were pregnancy, previous surgery related to the UC (e.g., colectomy), active disease (partial Mayo score > 3), gastrointestinal infection or taking antibiotics 2 weeks before starting the study or refusing to comply with the study requirements. Upon study initiation, a lifestyle interview was completed to assess physical activity, supplement use, food aversions, and other food beliefs, control for baseline habits, and account for any response bias.

Using the randomization ratio of 1:1 in block sizes of two, participants were randomly assigned to follow the MDP or Canada’s Habitual Diet Pattern (CHD) for 12 weeks. The allocation list was prepared by a nurse not involved in the trial. Participants were not aware of their allocation until informed consent was completed. Due to the need for the dietitians to provide nutrition education, group assignments were not blinded; however, participants and their healthcare providers remained blinded.

Due to challenges with recruitment, as a result of COVID-19, our protocol was amended, and we opened our recruitment criteria to include participants that were prescribed biologics (adalimumab, infliximab) and recruited from sites outside of the Okanagan Valley (Vancouver, Victoria, BC and Edmonton AB, Canada). Recruitment continued for an additional year (September 2020 to September 2021) with the expanded criteria; however, a decision was made to stop the trial early due to continual challenges with COVID-19 and backlogs in patient care resulting in a lack of dedicated clinical research time for the recruiting gastroenterologists. The primary analysis was intention-to-treat and involved all participants that completed the study requirements leaving a final sample size for analysis of 28 participants (MDP *n* = 15, CHD *n* = 13) (Figure 1A). No adverse events occurred.

### Diet intervention

The participants randomized to the MDP received a series of one-on-one online coaching sessions from a Master’s trained registered dietitian (RD; NH) with expertise in nutrition and IBD. The participants were taught how to adapt their diet based on the Mediterranean Diet Pyramid: (10) ≥ three servings (125 g/serving) per day of canned, fresh or frozen vegetables, ≥ three servings (125 g/serving) per day of canned, fresh or frozen fruit (avoiding juices), two servings/day of full-fat dairy products, wholegrain cereals, two servings of legumes (40 g/serving) per week, two servings of fatty fish (150 g/serving) per week; low consumption of red meat (<two servings per week), avoidance of refined grains, processed baked goods, processed meats, sweets, soft drinks and fresh juices, fast foods, and pre-cooked meals. Optionally, in alcohol drinkers, moderate consumption of red wine or beer (4 ounces/day) was also allowed. The key focus was a shift in dietary fat consumption with the recommendation of including at least 15 ml of MUFA-rich fat (olive oil or avocado) per meal, 25–30 grams of MUFA-rich nuts per day (almonds, hazelnuts, pecans, and macadamia nuts) and instructed to limit intake of foods rich in n-6 PUFA (corn, safflower, sunflower oil, margarine, prepared salad dressings, and highly processed foods) to achieve an intake of 35% total fat, 15% MUFA (mainly from olive oil), 13% SFA, 6% PUFA, and a high MUFA/SFA ratio, reflective of the intake of fat in Mediterranean countries (11). Resources such as MDP-specific recipes (16), MDP 4-week meal plan (16), MDP food lists and MDP snack ideas were used to support the nutrition intervention. The nutrition intervention was delivered online at week 1 (baseline-1 h), week 3 (30 min), week 6 (30 min), week 9 (30 min), and week 12 (end of study - 1 h) and via e-mail as requested. All participants completed the web-based Automated Self-Administered 24-h Canada 2016 Dietary Assessment Tool (ASA-24^®^-Canada-2016) (17) immediately before each visit with the dietitian. When participants logged on to ASA-24 they were asked to report all food and drink consumed in the previous 24 h. Portion sizes and nutrients were downloaded from the ASA-24 researcher website and compared with the Dietary Reference Intakes (DRI) (18) and the MD diet pyramid (10) to ensure both macronutrient and micronutrient adequacy. The CHD participants met with the dietitian at a similar intervention intensity to the MDP and received a nutritional analysis comparing their intakes to the DRI’s, however, they were not provided tailored dietary advice.

### Outcome and data collection

#### Dietary adherence

The Mediterranean Diet Serving Score (MDSS) assessed adherence to the MDP (19). The MDSS is a validated instrument based on the Mediterranean Diet Pyramid (10) which assesses total MDP adherence and allows for the assessment of individual food groups. Participants with an intake within the recommended servings for each food group are awarded a score of 3, 2, or 1 point. A score of 3 is awarded for food groups that should be consumed at every meal [fruit, vegetables, olive oil, cereals (whole grain bread, rice, pasta, and breakfast cereals)], followed by a score of 2 for foods that should be consumed daily (dairy products and nuts), and finally, a score of 1 for foods that are consumed less often (potatoes, legumes, eggs, fish, white meat, red meat, sweets, and fermented beverages). A score of 0 is given if the eating guidelines are not followed. The MDSS score ranges from 0 to 24 points, with greater than 13.5 points indicating adherence to the MDP. Moreover, the ASA24^®^-Canada 2016 (17) data was used to validate the MDSS and enhance compliance.

The Healthy Eating Index-2015 (HEI-2015) scoring method was used to measure the diet quality of both the MDP and CHD using the methods previously described (20). Briefly, the HEI-2015 score ranges from 0 to 100, with higher scores meaning a higher-quality diet. The HEI-2015 consists of 13 components: total fruit, whole fruit, total vegetables, greens and beans, total protein food, seafood and plant proteins, whole grain, dairy, fatty acid ratio, refined grain, sodium, and added sugar, and saturated fats. Six items (total fruit, whole fruit, total vegetables, greens and beans, total protein food, and seafood and plant proteins) score five points each, and the other seven items score 10 points (20). Food consumption was obtained from the median intakes of the ASA-24 (24-h recalls) and translated to cup and ounce equivalents to calculate the HEI-2015 scores.

Several clinical trials have used the HEI-2015 to investigate the relationship between dietary quality in other chronic diseases, such as cancer or cardiovascular disease. However, to our knowledge, there is a lack of studies that have examined the overall diet quality in UC. Thus, the HEI-2015 was included as a study outcome after the trial commenced to better quantify the quality of the dietary pattern in our patient population.

#### Quality of life assessment

The Short Inflammatory Bowel Disease Questionnaire (SIBDQ) is a validated questionnaire used to assess the quality of life in IBD (21). The SIBDQ includes 10 questions of four domains that assess bowel symptoms, social function, systemic function, and emotional status. The score for each question ranges from 1 to 7, with 1 indicating significant impairment and 7 indicating no impairment in quality of life. The total score ranges from 10 to 70 points, with higher scores indicating good quality of life.

### Stool long chain fatty acid analysis

Direct-injection gas chromatography was used to quantify long-chain fatty acids from stool. Fatty acids were extracted by using a combined extraction and methylation protocol. In summary, 200 μl QIAzol and 50 μl chloroform was added to 500 mg of homogenized stool. The stool was centrifuged for 15 min at 13,000 rpm. The supernatant was collected, followed by adding 1.2 ml of hexane and 1.2 ml of boron trifluoride-methanol solution 14% in methanol, Sigma Aldrich, Catalog #B1127. Samples were heated at 80°C for 2 h. Next, 2 ml of deionized water was added to the samples and centrifuged at 1,400 rpm for 2 min, and the top hexane layer was removed. The supernatant was injected into a Trace 1,300 Gas Chromatograph, equipped with a flame-ionization detector, with AI1310 autosampler (Thermo Fisher Scientific, Waltham, MA, USA) in splitless mode. A fused silica Rtx-WAX (Restek, Bellefonte, PA, USA) column 30 m × 0.32 mm i.d. coated with 0.5 μm film thickness was used. Helium was supplied as the carrier gas at a flow rate of 1.8 ml/min. The initial oven temperature was 100°C, maintained for 5 min, raised to 240°C at 4°C/min, then held for 15 min. The flame-ionization detector and the injection port temperature were 280°C and 250°C, respectively. The flow rates of hydrogen, air and nitrogen as makeup gas were 35, 350, and 30 ml/min, respectively. Peak areas and retention times were then calculated using Chromeleon 7 software (Bannockburn, IL, USA) and compared against standards (Supelco 37 Component FAME Mix, Sigma Aldrich, Catalog #CRM47885). Values were expressed as a percent of total fatty acid derived from the area of a single fatty acid peak/total area of peaks.

### Outcome measures

The primary outcome was the proportion of participants reaching high adherence (>16 points) as measured by the MDSS from baseline to week 12. Additional analyses included changes in diet quality (HEI-2015), quality of life (SIBSQ), and the nutritional adequacy of the diet [compared to Recommended Dietary Allowance (RDA) or Adequate Intake (AI)] from baseline to 12 weeks.

### Statistical analysis

The statistical package GraphPad Prism (Version 9.3.0) was used for analysis. Continuous data are presented as the median and interquartile range (IQR), whereas categorical data are presented as absolute value and percentage. The normality of data distribution for continuous variables was assessed with a D’Agostino–Pearson test. Fisher’s exact test was used to test differences between groups of categorical data. The Wilcoxon matched-pairs signed-rank test was used for paired data to assess pre- and post-dietary intervention. The Mann–Whitney *U*-test was used for the comparison between groups. *P*-values of < 0.05 were considered statistically significant.

## Results

### Participant flow and baseline characteristics of the participants

A total of 80 patients with IBD were screened for their eligibility with a final study sample of 28 participants (MDP, *n* = 15 vs. CHD, *n* = 13). The study consort flowchart is shown in Figure 1A. There was no significant difference in the baseline characteristics of participants (Table 1). All participants entered the study in clinical remission, defined as a partial Mayo score between 0 and 2. A large majority of the participants reported food intolerance (47% in MDP and 77% in CHD) and were deemed at nutrition risk according to the SaskIBD-NR screening tool (53% in MDP and 53% in CHD; *p* = 0.70). The most common food intolerances reported in the study cohort were dairy (36%), gluten (21%), red meat (21%), and vegetables (18%).

**TABLE 1:** Baseline characteristics of the study participants.

Baseline characteristics	MDP (*n* = 15)	CHD (*n* = 13)	*P*-value
**Sex, n (%)**			
Male	6 (40)	4 (31)	0.71^a^
Female	9 (60)	9 (69)	
**Age, (years)**			
Median (IQR)	52 (21)	39 (24)	0.30^b^
Min-max	18–65	25–63	
**Smokers, n (%)**			
Yes	4 (27)	3 (23)	>0.99^a^
No	11 (73)	10 (77)	
**Physical activity (>150 min/week), n (%)**			
Yes	12 (80)	13 (100)	0.23^a^
No	3 (20)	0	
**BMI (kg/m^2^)**			
Median (IQR)	23 (11)	22 (8)	0.79^b^
Min-max	17–30	19–29	
**BMI classification, n (%)**			
Underweight (<18.5)	3 (20)	1 (8)	0.30^a^
Normal weight (18.5 – < 25)	6 (40)	9 (69)	
Overweight (25.0 – < 30.0)	5 (33)	3 (23)	
Obese (>30)	1 (7)	−	
Food intolerance, n (%)			0.25^a^
Yes	7 (47)	10 (77)	
No	8 (53)	3 (23)	
**SaskIBD-NR**			
Low risk (0)	7 (47)	5 (38)	0.70^a^
Medium risk (1)	7 (47)	8 (53)	
High risk (≥2)	1 (6)	−	
**Vitamin levels (Median, IQR)**			
Vitamin D (ng/ml)	87 (16)	96 (34)	0.21^b^
Vitamin B12 (pg/ml)	425 (281)	473 (407)	0.95^b^
**Disease duration, (years)**			
Median (IQR)	10 (29)	10 (32.5)	0.72^b^
Min-max	1–30	1–33	
Current medications, n (%)			0.55^a^
No medications	4 (27)	2 (15)	
5-aminosalicylic acid	8 (53)	10 (77)	
Biologic only	2 (13)	−	
Thiopurine + Biologic	1 (7)	1 (8)	
**Partial Mayo score**			
Total score (range)	0 (0–2)	0 (0–1)	>0.99^b^

MDP, Mediterranean diet pattern; CHD, Canada’s Habitual Diet Pattern; IQR, Interquartile range; BMI, body mass index; Sask IBD-NR, Saskatchewan Inflammatory Bowel Disease Nutrition Risk (22). ^a^Fisher’s exact test. ^b^Mann-Whitney test. Statistically significant difference *p* < 0.05.

### Quality of life

No statistical differences in total QOL scores from baseline to week 12 were seen (Table 2); however, between-group analyses by the SIBDQ domain revealed the bowel domain was the most responsive in the MDP. Further sub-group analysis of the bowel domain showed a reduction in the passing of large amounts of gas (*p* = 0.01) and improvements in tenesmus (*p* = 0.03).

**TABLE 2:** Mean change in the health-related quality of life scores from baseline to week 12 demonstrates improvements in bowel symptoms for the Mediterranean diet pattern.

SIBDQ domains	MDP^a^ (*n* = 15)			CHD^a^ (*n* = 13)			Between groups^b^ (Week 12)
	**Mean **Δ****		***P*-value**	**Mean **Δ****		***P*-value**	***P*-value**
Bowel symptoms	0.84		0.0004	0		0.73	0.41
Systemic symptoms	0		0.86	−0.27		0.57	0.80
Emotional function	−0.31		0.30	0.82		0.03	0.95
Social function	−0.03		0.75	−0.04		0.49	0.03
Total SIBDQ score	58	62	0.30	58	60	0.21	0.85
(Median, IQR)	(10)	(9)		(13)	(9)		

MDP, Mediterranean diet pattern; CHD, Canada’s Habitual Diet Pattern; IQR, interquartile range; SIBDQ, Short inflammatory bowel disease questionnaire. ^a^Wilcoxon-signed rank test, ^b^Mann-Whitney test. Statistically significant difference *p*-value < 0.05.

### Mediterranean diet adherence

Adherence to the MDP was assessed using the MDSS for the 28 participants at baseline and week 12 (Table 3). No significant differences in MDSS were detected at baseline between the MDP vs. CHD (*p* = 0.19). At week 12, the MDP had a significantly higher total MDSS (median 19, range 12–23) vs. the CHD (median 14, range 5–21, *p* = 0.01), indicating the MDP had better adherence. A high level of adherence (total score of 16–24) was achieved in the MDP (*n* = 10) vs. CHD (*n* = 3; *p* = 0.030). There were no changes in adherence to the MDSS from baseline to week 12 in the CHD group (Supplementary File 1). After the 12 weeks, the MDP consumed more fruit (87%), vegetables (67%), dairy (80%), legumes (80%), olive oil (73%), fish (93%), and consumed fewer sweets (67%) (Table 4).

**TABLE 3:** The Mediterranean diet serving score for the Mediterranean diet pattern and Canada’s habitual diet at week 12.

MDSS parameter (n, %)	MDP (*n* = 15)	CHD (*n* = 13)	Between groups^a^ (Week 12)
Fruit, 1–2 servings per main meal	13 (87)	6 (46)	0.04
Vegetables, ≥ 2 servings per main meal	10 (67)	7 (54)	0.70
Dairy products, ≥ 2 servings per day	12 (80)	8 (62)	0.41
Cereals, 1–2 servings per meal	11 (73)	8 (62)	0.69
Legumes, ≥ 2 servings per week	12 (80)	5 (38)	0.05
Potatoes, 3 servings per week	13 (87)	9 (69)	0.37
Olive oil, 1–15 ml serving or more per main meal	11 (73)	3 (23)	0.02
Nuts, 1–2 servings or more/day	11 (73)	7 (54)	0.43
Eggs, 2–4 servings per week	12 (80)	9 (69)	0.67
Fish, ≥ 2 servings per week	14 (93)	11 (85)	0.58
White meat, 2 servings per week	12 (80)	10 (77)	>0.99
Red meat, < 2 servings per week	13 (87)	10 (77)	0.64
Sweets < 2 servings per week	10 (67)	5 (38)	0.03
Fermented beverages, 1–2 glass/day	7 (47)	4 (31)	0.46
Total MDSS score (n, range)	19 (12–23)	14 (5–21)	0.01
Adherence to MDP (Total score)			
Low, score 0–8 (n, %)	0	3 (23)	0.09
Moderate, score 9–15	5 (33)	7 (54)	0.45
High, score 16–24	10 (67)	3 (23)	0.03

MDSS, Mediterranean diet serving score; CHD, Canada’s Habitual Diet Pattern; MDP, Mediterranean diet pattern. ^a^Mann-Whitney test. Statistically significant difference *p*-value < 0.05.

**TABLE 4:** The Mediterranean diet serving score for the Mediterranean diet pattern at baseline and week 12.

MDSS parameter (n, %)	MDP baseline (*n* = 15)	MDP week 12 (*n* = 15)	Between groups^a^ (*n* = 30)
Fruit, 1–2 servings per main meal	6 (40)	13 (87)	0.02
Vegetables, ≥ 2 servings per main meal	3 (20)	10 (67)	0.03
Dairy products, ≥ 2 servings per day	4 (27)	12 (80)	0.01
Cereals, 1–2 servings per meal	8 (53)	11 (73)	0.45
Legumes, ≥ 2 servings per week	3 (20)	12 (80)	0.003
Potatoes, 3 servings per week	11 (73)	13 (87)	0.65
Olive oil, 1–15 ml serving or more per main meal	14 (93)	11 (73)	0.001
Nuts, 1–2 servings or more/day	5 (33)	11 (73)	0.07
Eggs, 2–4 servings per week	8 (53)	12 (80)	0.25
Fish, ≥ 2 servings per week	5 (33)	14 (93)	0.002
White meat, 2 servings per week	11 (73)	12 (80)	>0.99
Red meat, < 2 servings per week	12 (80)	13 (87)	>0.99
Sweets, < 2 servings per week	3 (20)	10 (67)	0.03
Fermented beverages, 1–2 glass/day	7 (47)	7 (47)	>0.99
Total MDSS score (n, range)	9 (6–11)	19 (14–22)	0.0001
Adherence to MDP (Total score)			
Low, score 0–8 (n, %)	5 (33)	0	0.04
Moderate, score 9–15 (n, %)	9 (60)	5 (33)	0.27
High, 16–24 (n, %)	1(7)	10 (67)	0.002

MDSS, Mediterranean diet serving score; MDP, Mediterranean diet pattern. ^a^Wilcoxon-signed rank test. Statistically significant difference *p*-value < 0.05.

### Assessment of diet quality (HEI-2015)

**FIGURE 2:**
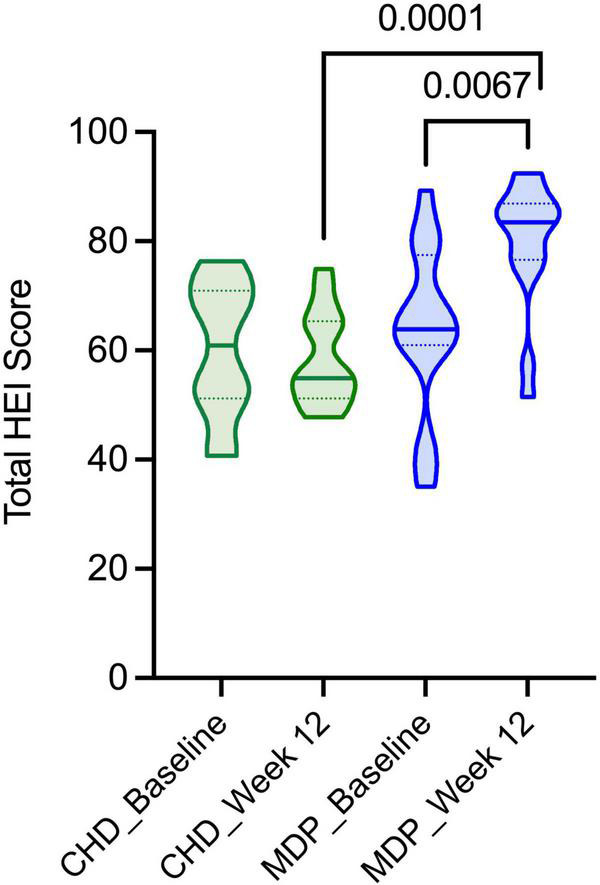
The HEI-2015 scores for the Mediterranean diet pattern and Canada’s Habitual Diet Pattern. Consumption of the MDP resulted in improved diet quality compared to the CHD at week 12 and improved following the diet intervention (baseline to week 12).

Figure 2 shows the differences in HEI-2015 scores for the MDP and CHD at baseline and week 12. Participants following the MDP increased the quality of their diet (HEI-2015 score) significantly from baseline to week 12 (64–80; *p* = 0.007) and had a significantly higher HEI-2015 at week 12 compared to the CHD (80 vs. 58, respectively; *p* < 0.0001). Table 5 shows the individual dietary components of the HEI-2015 for the MDP and CHD at baseline and week 12. Participants following the MDP consumed significantly more total fruit (*p* = 0.05), vegetables (*p* = 0.03), and less added sugars (*p* = 0.04) from baseline to week 12, whereas no significant changes were seen in the CHD. Fatty acids (PUFA + MUFA/SFA; max score 10) changed in both groups; however, the MDP saw the most considerable within-group improvement (5.6–10.0; *p* = 0.004). The change in MDP is from the increased MUFA intake of nuts and fish confirmed by the MDSS (Table 4). Comparing the MDP and CHD at week 12 demonstrated that the MDP increased intake of total fruit (*p* = 0.05), greens and beans (*p* = 0.02), whole grains (*p* = 0.001) with a concomitant reduction in refined grains (*p* = 0.01), sodium (*p* = 0.03), and added sugars (*p* = 0.003). The MDP vs. CHD saw a trend for increased consumption of whole fruit (*p* = 0.07) and saturated fat (*p* = 0.06). The MDSS showed increased dairy product intake (full-fat) in the MDP group, which may account for the increased saturated fat (Table 4).

**TABLE 5:** Median HEI-2015 component scores for the Mediterranean diet pattern and Canadian habitual diet pattern at baseline and week 12, with higher scores reflective of better diet quality.

HEI-2015 dietary component (Maximum score)	MDP^a^ (*n* = 15)			CHD^a^ (*n* = 13)			Between groups^b^ (Week 12)
	**Baseline**	**Week 12**	***P*-value**	**Baseline**	**Week 12**	***P*-value**	***P-*value**
Total fruit^c^ (5)	4.0 (3.5)	5.0 (4.3)	0.05	3.3 (4.0)	5.0 (4.0)	0.50	0.05
Whole fruit^d^ (5)	5.0 (5.0)	5.0 (1.9)	0.11	5.0 (5.0)	5.0(5.0)	0.60	0.07
Total vegetables (5)	2.9 (5.0)	5.0 (3.3)	0.03	3.3 (4.3)	5.0 (4.0)	0.17	0.78
Greens and beans (5)	0 (5.0)	5.0 (5.0)	0.26	2.2(4.3)	0 (4.0)	0.31	0.02
Whole grains (10)	2.0 (10.0)	5.7 (10.0)	0.20	0.15 (9.8)	0.62 (4.9)	0.28	0.001
Dairy (10)	6.0 (9.7)	4.5 (9.2)	0.97	7.3 (9.9)	3.3 (10)	0.24	0.49
Total protein foods (5)	5.0 (4.7)	5.0 (4.4)	0.25	5.0 (3.5)	5.0 (2.0)	0.16	0.41
Seafood and plant proteins (5)	3.9 (5.0)	5.0 (5.0)	0.23	4.1 (5.0)	5.0 (5.0)	0.76	0.29
Fatty acid ratio^e^ (10)	5.6 (10)	10.0 (8.0)	0.004	2.8 (10.0)	6.38 (8.9)	0.04	0.22
Saturated fat^f^ (10)	7.3 (10.0)	8.0 (10.0)	0.33	4.8 (9.0)	5.6 (9.3)	>0.99	0.06
Refined grains (10)	9.6 (7.1)	10.0 (4.9)	0.06	7.8 (10.0)	8.5 (10.0)	0.85	0.01
Sodium (10)	8.5 (10.0)	10.0 (10.0)	0.28	8.0 (10.0)	6.3 (10.0)	0.57	0.03
Added sugars (10)	10.0 (10.0)	10.0 (1.4)	0.04	9.0 (8.8)	8.7 (7.7)	>0.99	0.003
Total score	64	83	0.007	61	55	0.59	<0.0001
Median (IQR)	(61–89)	(77–92)		(51–76)	(51–75)		

HEI-2015, healthy eating index-2015; MDP, Mediterranean diet pattern; CHD, Canada’s Habitual Diet Pattern; IQR, interquartile range. ^a^Wilcoxon signed rank test. ^b^Mann-Whitney test. ^c^Total fruit includes 100% fruit juice. ^d^Fruit excludes fruit juice. ^e^Total MUFA + Total PUFA/Total SFA. ^f^Saturated fat less than or equal to 8%. Data presented as median (IQR). Statistically significant difference *p*-value < 0.05.

### Assessment of nutritional adequacy

**FIGURE 3:**
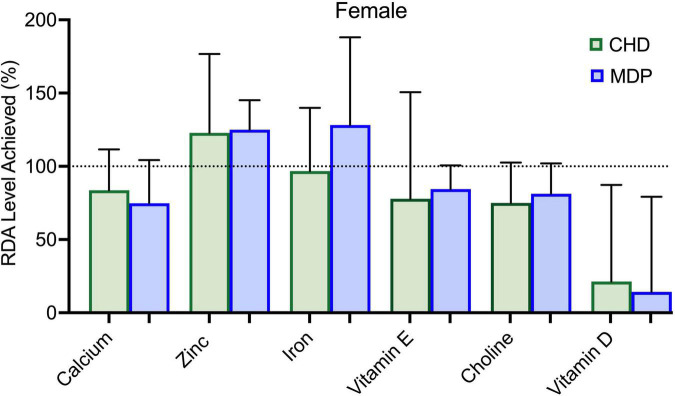
Micronutrient intake of females compared to RDA for the Mediterranean diet pattern (MDP) and Canada’s Habitual Diet Pattern (CHD).

**FIGURE 4:**
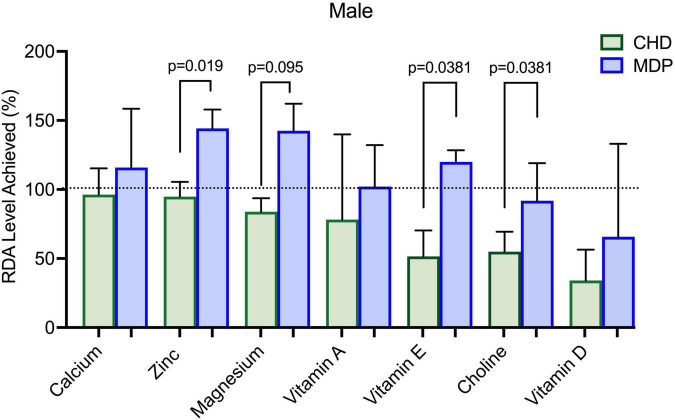
Micronutrient intake of males compared to RDA for the Mediterranean diet pattern (MDP) and Canada’s Habitual Diet Pattern (CHD).

Figures 3, 4 summarizes the micronutrient adequacy of the CHD and the MDP at week 12 of the diet intervention. Male participants following the CHD did not achieve the recommended dietary allowance (RDA) levels for calcium, zinc, magnesium, vitamin A, vitamin E, vitamin D, and choline. In contrast, the male MDP participants did not achieve the RDA/AI levels for vitamin D, choline, and fiber. Female participants following the CHD did not meet RDA levels for calcium, iron, vitamin A, vitamin E, and the AI for choline. In contrast, the female MDP participants did not achieve RDA/AI intakes for calcium, iron, vitamin D and E, and choline. Examination of all other macro-and micronutrients indicated that both diets adequately met the RDA’s (Supplementary Files 2–5).

### Stool long-chain fatty acid analysis

**FIGURE 5:**
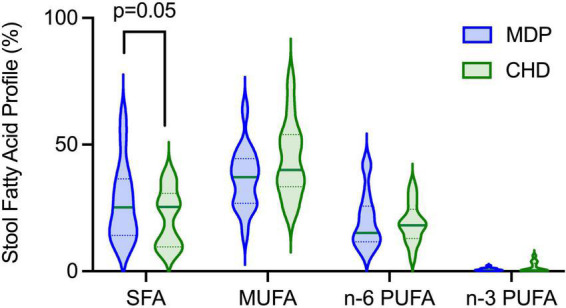
The MDP had increased stool excretion of saturated fatty acids at week 12.

Gas chromatography was used to determine the concentrations of long chain fatty acids excreted in the stool with no differences observed in MUFA, n-6 PUFA or n-3 PUFA excretion between diet groups (Figure 5). No sex differences were observed (Supplementary File 6). The MDP showed a significant increase in SFA excretion, with the highest increase seen in palmitic acid (C16:0), indicating that the consumption of the MDP components could influence SFA metabolism.

## Discussion

In this cohort of adult participants with quiescent UC, implementing the MDP was associated with improved bowel symptoms, nutritional adequacy, and quality of the diet compared to participants following CHD. The bowel symptoms that improved in the participants following the MDP were gas and tenesmus, despite the introduction of foods that were previously avoided due to intolerance.

Dietary patterns can be evaluated using different a priori indexes, with some of the most popular being the MDSS and HEI-2015. We demonstrate high compliance, with 67% of participants assigned to the MDP scoring 16 or greater on the MDSS. We also saw that the median value of the HEI-2015 improved from 64 to 83 (*p* < 0.007) in the MDP, indicative of a better-quality diet. High adherence to recommended guidelines was observed for eating fruit (87%), vegetables (67%), dairy (80%), legumes (80%), olive oil (73%), fish (93%), and consumption of fewer sweets (67%) with no reported increase in symptoms, in fact, participants reported fewer symptoms. Our results contrast other studies, where participants struggled to adhere to the MDP (13, 23). We attribute this success to intense counseling provided by a Master’s trained Registered Dietitian with expertise in IBD and nutrition, who provided continuous support through nutrition assessment and education on the MDP. Additionally, specific guidance on portion sizes provided to the MDP participants was a means for participants to enhance their diet quality. We believe these supports made it easier for the participants to adhere to the diet.

To further confirm compliance, we examined the stool’s composition of long-chain fatty acids. We found significantly higher SFA excretion in the stool in the MDP than in the CHD, which can partially be explained by dietary intake. Fatty Acid ratio scores (HEI-2015) were significantly higher in the MDP at week 12 from baseline. The Fatty Acids ratio score is a composite score representing the ratio of unsaturated fatty acids to SFAs. The fat composition of higher Fatty Acids scores can reflect greater intakes of monounsaturated and/or polyunsaturated fats, lower intakes of saturated fats, or both. In this study, the fat composition of Fatty Acids scores reflects greater intakes of olive oil monounsaturated fats (primarily olive oil), supported by the MDSS, as saturated fat intake (HEI-2015) did not change. To further support these findings, we have shown in a rodent model of chronic colitis that various fatty acids impact metabolism and inflammation differently (24). For example, in an inflammatory state, preferences for fatty utilization change leading to alterations in absorption and excretion. We conclude that the improved fatty acid composition of the diet influenced SFA excretion but also could suggest that the excretion of fatty acids could be influenced by the participant’s inflammatory state. Further work in human studies is needed to confirm these findings.

Although our results suggest high compliance with the MDP, some nutrients did not meet the RDA/Adequate intake levels, which is important as patients with UC are at nutrition risk for micronutrient deficiencies (25, 26). Male participants that followed the MDP were low in choline intake, and the females following the MDP remained low in calcium, vitamin E, and choline intake. Although female participants increased their intake of dairy products, it was not sufficient to meet the RDA for calcium. Future dietary counseling using the MDP should focus on alternate ways to meet calcium requirements to ensure nutrition adequacy. Concerning dietary choline, both groups of participants in this study had intakes well below the Adequate Intake for Canadians. The importance of this “essential” nutrient cannot be understated, as it has essential functions across the lifespan, with deficiencies linked to liver disease, cognitive dysfunction, and neurological disorders (27). Due to its important role in synthesizing phospholipids, lipid transport proteins, and methylation reactions (27), choline is needed for metabolism, maintaining liver health, and reducing homocysteine levels. Until recently, little attention has been paid to dietary choline intake and its association with IBD. A recent *in vivo* study in an infectious model of colitis (*Citrobacter rodentium*-induced colitis) found that insufficient dietary choline aggravated the severity of colitis and was crucial in maintaining intestinal homeostasis (28). Also, choline deficiencies have been linked to an increased risk of non-alcoholic fatty liver disease in the IBD population (29). It is vital to examine choline deficiency given the accelerated food trends toward plant-based diets/veganism and environmental sustainability which could further negatively impact choline intake/status. Restricted intakes of dairy, eggs, and animal protein, which are high bioavailable sources of choline, will affect choline intake and status. Further study of dietary choline intakes in the IBD population is worthy of consideration, primarily due to the emerging link between microbial metabolism, its potential role in inflammation, and maintenance of intestinal homeostasis.

Previous studies that report that the average fiber intake in patients with quiescent UC ranges from 9 to 13.5 grams/day (30). Fiber intake in the females following the MDP increased significantly. The males in both groups could not achieve the recommended RDA intake for fiber (goal 38 grams/day). Yet, despite not meeting these guidelines, the males in the MDP were able to tolerate 24 grams/day without a reported increase in symptoms.

Today, there is no standard method to assess overall diet, and multiple approaches are needed to examine dietary intakes, with each approach having its advantages and disadvantages. We are seeing a shift in current dietary recommendations from focusing on individual foods/nutrients to examining whole eating patterns to account for inter-relations of food choices, represent the cumulative exposure to different diet components, and the synergistic effects of a diet pattern on health (31). A strength of this study is that we examined both individual nutrients (RDA/AI) (18), and dietary patterns (HEI-2015 and MDSS) to examine the study participants’ diet to provide a holistic view of this cohort of participants with UC. The results of our study stimulate further interest in the relationships between the influence of dietary patterns, nutrient adequacy, and disease management in IBD. In addition, identifying dietary patterns can pave the way for future understanding of the link between overall diet quality and the intestinal microbiome in patients with UC.

This study has some limitations; in particular, it is underpowered due to its small sample size. Its small sample size limits the generalizability of this work. The strengths include using validated questionnaires, using a Registered Dietitian-Scientist with expertise in IBD and evidence-based nutrition, and using multiple-pass 24-h recall records, which are considered accurate and precise for measuring dietary intake data in clinical practice (32). The diet had an excellent uptake by participants, and several participants continued the MDP after completing the study. Although time commitment was not measured in our research, starting the MDP relies upon a considerable input of time and energy of the participants themselves, especially in the early phases; however, once the participants developed a routine, less time was spent planning their diet.

## Conclusion

The MDP involves including various pleasurable food and drinks vs. strict dietary exclusion, allowing for lifelong healthy eating habits. Exclusion diets are highly restrictive, leading to potentially unhealthy relationships with food and can trigger disordered eating, which is highly prevalent in the IBD population (33). Patients with UC can adhere to and tolerate the MDP and achieve improved diet quality with reduced bowel symptoms compared to CHD. Increased adherence to the MDP can be achieved through appropriate support such as nutrition education and counseling by a Registered Dietitian. Overall, this data supports that the MDP can be recommended as a remission diet to UC patients to help support disease management alongside front-line therapy drugs.

## Data availability statement

The datasets presented in this study can be found in online repositories. The names of the repository/repositories and accession number(s) can be found below: data repository at NH (2022), “Dietary compliance with the Mediterranean diet pattern in a randomized clinical trial of patients with quiescent Ulcerative Colitis,” Mendeley Data, V1, doi: 10.17632/2ffyvrdd97.1.

## Ethics statement

The studies involving human participants were reviewed and approved by the University of British Columbia Clinical Research Ethics Board (H16-03300). The patients/participants provided their written informed consent to participate in this study. The manuscript was prepared according to the Consolidated Standards of Reporting Trials Statement (http://www.consort-statement.org) (Supplementary File 7) and the TIDieR Checklist (Supplementary File 8).

## Author contributions

NH and DG designed the research. DG, NH, RS, SS, KJ, and LD recruited the participants and provided expertise. NH conducted the study. NH, RS, AD-H, and JY analyzed the data with input from DG. NH wrote the original draft of the manuscript. RS, DG, KJ, SS, SG, and LD reviewed and edited the manuscript. DG supervised, provided resources, and funding for this project. All authors have read and approved the final manuscript.
