# Supplementary material for: Dietary adherence to the Mediterranean diet pattern in a randomized clinical trial of patients with quiescent ulcerative colitis
Source: Front Nutr. 2022 Dec 21;9:1080156. doi: 10.3389/fnut.2022.1080156 (PMC9812483; doi:10.3389/fnut.2022.1080156)
Supplement: Supplementary file 1 [file Data_Sheet_1.DOCX]

**On-line Additional Files**

**Dietary compliance with the Mediterranean diet pattern in a randomized clinical trial of patients with quiescent Ulcerative Colitis**

**Authors**

Haskey N^1^, Shim RK^2^, Davidson-Hunt A^1^, Ye J^3^, Singh S^4^, Dieleman LA^5^, Jacobson K^6^, Gibson DL^1,7, #^

^1^Department of Biology; University of British Columbia - Okanagan Campus; Kelowna, British Columbia, V1V 1V7, Canada

^2^Department of Public Health Sciences, The Dalla Lana School of Public Health, University of Toronto, Toronto, Ontario, Canada

^3^ Diabetes Center, University of California San Francisco, San Francisco, USA

^4^Department of Medicine, Division of Gastroenterology, University of British Columbia, Vancouver, British Columbia, Canada

^5^Department of Medicine, Division of Gastroenterology, University of Alberta, Edmonton, Alberta, Canada

^6^Department of Pediatrics, Division of Gastroenterology, Hepatology and Nutrition and British Columbia Children's Hospital Research Institute, University of British Columbia, Vancouver, BC, Canada

^7^Department of Medicine; University of British Columbia - Okanagan Campus, Kelowna, British Columbia, V1V 1V7, Canada

| **Title** | **Page** |
| --- | --- |
| Additional File 1: Mediterranean Diet Serving Score for Canada’s Habitual Diet Pattern (CHD) at baseline and week 12 | 3 |
| Additional File 2. Median nutrient intakes of the female participants on the MDP and CHD at baseline and week 12 | 4-5 |
| Additional File 3. Fatty acid composition of the diets calculated as a percentage of total calories (females) | 5 |
| Additional File 4. Median nutrient intakes of the male participants on the MDP and CHD at baseline and week 12 | 6-7 |
| Additional File 5. Fatty acid composition of the diets calculated as a percentage of total calories (males) | 7 |
| Additional File 6. Fatty acid excretion in the stool (male and females) | 8 |
| Additional File 7. Consort 2010 Check-list | 9 |
| Additional File 8. TIDierR Check-list | 10 |

**Table of Contents**

Additional File 1. Mediterranean Diet Serving Score for Canada’s Habitual Diet Pattern (CHD) at baseline and week 12

| MDSS Parameter (n, %) | CHD Baseline  (n=13) | CHD  Week 12  (n=13) | Between Groups^a^  (n=26) |
| --- | --- | --- | --- |
| Fruit, 1-2 servings per main meal  Vegetables,≥ 2 servings per main meal  Dairy products, ≥ 2 servings per day  Cereals, 1-2 servings per meal  Legumes, ≥ 2 servings per week  Potatoes, 3 servings per week  Olive oil, 1-15 ml serving or more per main meal  Nuts, 1-2 servings or more/day  Eggs, 2-4 servings per week  Fish, ≥ 2 servings per week  White meat, 2 servings per week  Red meat, < 2 servings per week  Sweets, < 2 servings per week  Fermented beverages, 1-2 glasses/day  Total MDSS score (n, range)  Adherence to MDP (Total Score)  Low, score 0-8 (n, %)  Moderate, score 9-15 (n, %)  High, 16-24 (n, %) | 4 (31)  9 (69)  5 (38)  8 (62)  5 (38)  13 (100)  1 (8)  5 (38)  10 (77)  7 (54)  5 (38)  8 (62)  5 (38)  2 (15)  7 (7-14)  4 (31)  7 (54)  2 (15) | 6 (46)  7 (54)  8 (62)  8 (62)  5 (38)  9 (69)  3 (23)  7 (54)  9 (69)  11 (85)  10 (77)  10 (77)  5 (38)  4 (31)  14 (5-21)  3 (23)  7 (54)  3 (23) | 0.67  0.69  0.44  >0.99  >0.99  0.10  0.59  0.70  >0.99  0.20  0.11  0.67  >0.99  0.65  0.30  >0.99  >0.99  >0.99 |

Abbreviations: MDSS, Mediterranean diet serving score; CHD, Canada’s Habitual Diet Pattern. ^a^Wilcoxon-signed rank test. Statistically significant difference p-value < 0.05.

Additional File 2. Median nutrient intakes of the female participants on the MDP and CHD at baseline and week 12

|  |  | **MDP**  **(n=9)** | | | **CHD**  **(n=9)** | | |  |
| --- | --- | --- | --- | --- | --- | --- | --- | --- |
| ***Female***  **(Median, IQR)** | **RDA/AI** | **Baseline** | **Week 12** | **P value within group^a^** | **Baseline** | **Week 12** | **P value within groups^a^** | **P value between intervention groups^b^** |
| Energy  (kcal/day) | 20-25 kcal/kg | 1959 (489) | 1921 (1120) | 0.43 | 1924 (836) | 1773  (819) | 0.88 | 0.45 |
| Carbohydrates (g/day) | 130 | 245  (127) | 160 (124) | 0.43 | 69  (177) | 57.8  (147) | 0.36 | 0.90 |
| Protein  (g/day) | 46 | 88.4 (46.2) | 71.9 (34.8) | 0.38 | 69.0 (20.1) | 71.3  (36.1) | 0.16 | 0.40 |
| Total fat  (g/day) | ND | 82.5 (31.8) | 76.8 (19.9) | 0.85 | 79.8 (58.1) | 76.5  (20) | >0.99 | 0.72 |
| Saturated fat (g/day) | ND | 25.2  (19.9) | 21.9  (4.4) | 0.63 | 26.8  (13.4) | 19.9  (5.6) | 0.65 | 0.90 |
| MUFA  (g/day) | ND | 33.3  (18.3) | 30.1  (35.7) | 0.77 | 28.9  (36.9) | 24.6  (16) | 0.65 | 0.97 |
| n-6 PUFA  (g/day) | 11  -12* | 14.0  (8.2 | 14.2  (7.8) | >0.99 | 12.0  (13.9) | 13.9  (11.8) | 0.65 | >0.99 |
| n-3 PUFA  (g/day) | 1.1* | 1.89  (1.5) | 2.20  (2.8) | 0.85 | 1.73  (2.3) | 1.59  (1.3) | 0.91 | 0.72 |
| Total fibre  (g/day) | 21  -25* | 22.4 (16.4) | 31.9 (12.2) | 0.04 | 22.7  (9.2) | 30.9  (23.7) | 0.13 | 0.97 |
| Sugars  (g/day) | <25% energy | 94.6 (114.5) | 84.7 (58.7) | >0.99 | 82.4 (73.9) | 72.3  (78.1) | 0.31 | 0.55 |
| Sodium  (mg/day) | 1300-1500* | 2885 (2081) | 1954 (1287) | 0.04 | 3015  (967) | 2103  (3437) | 0.31 | 0.37 |
| Calcium  (mg/day) | 1000-1200 | 810  (721) | 819 (756) | 0.77 | 1022  (550) | 690  (348) | 0.07 | 0.13 |
| Iron  (mg/day) | 18 | 12.5  (8.5) | 12  (6.3) | 0.05 | 12.3  (6.9) | 12.2  (8.4) | 0.57 | 0.66 |
| Magnesium  (mg/day) | 310  -320 | 350  (270) | 402 (196) | >0.99 | 379  (196) | 368  (192) | 0.82 | 0.36 |
| Phosphorus  (mg/day) | 700 | 1425 (715) | 1612  (798) | 0.85 | 1229 (420) | 1056  (325) | 0.20 | 0.01 |
| Zinc  (mg/day) | 8 | 9.5  (4.1) | 10.2 (4.3) | 0.85 | 9.5  (3.7) | 10.8  (5.7) | 0.82 | >0.99 |
| Copper  (mg/day) | 0.9 | 1.3  (1.1) | 1.4  (0.8) | 0.11 | 1.5  (0.5) | 1.6  (0.8) | 0.16 | 0.97 |
| Selenium  (μg/day) | 55 | 117  (66.1) | 92.5 (61.1) | 0.11 | 108  (34.4) | 67.8  (72.8) | 0.16 | 0.78 |
| Vitamin C  (mg/day) | 75 | 82  (123) | 101 (125) | 0.43 | 95  (101) | 151  (116) | 0.04 | 0.73 |
| Thiamin  (mg/day) | 1.1 | 1.6  (1.2) | 1.4  (0.5) | 0.13 | 1.1  (0.5) | 1.7  (0.7) | 0.43 | 0.14 |
| Riboflavin  (mg/day) | 1.1 | 2.2  (1.0) | 2.2  (0.9) | 0.09 | 2.0  (0.7) | 1.9  (0.6) | 0.73 | 0.67 |
| Niacin^c^  (mg/day) | 14 | 20  (12) | 20  (9) | 0.65 | 21  (15) | 21  (11) | 0.65 | 0.55 |
| Vitamin B6  (mg/day) | 1.3 | 1.6  (2.0) | 1.7  (1.0) | >0.99 | 1.8  (0.8) | 1.6  (1.4) | 0.38 | 0.86 |
| Folate^d^  (DFE/day) | 400 | 435  (156) | 455 (209) | 0.09 | 447  (296) | 520  (227) | >0.99 | 0.39 |
| Vitamin B12  (μg/day) | 2.4 | 3.9  (4.1) | 4.0  (3.1) | 0.36 | 3.6  (3.0) | 3.4  (2.6) | 0.50 | 0.60 |
| Vitamin A^e^  (μg/day) | 700 | 502  (813) | 1095 (556) | 0.16 | 529  (835) | 678  (1118) | 0.30 | 0.93 |
| Vitamin D  (μg/day) | 15 | 4.7  (9.2) | 2.1  (9.3) | 0.91 | 3.5  (5.6) | 3.2  (5.1) | 0.43 | 0.34 |
| Vitamin E (mg/day) | 15 | 7.6  (8.3) | 13  (4.0) | 0.09 | 14  (13.6) | 12  (14.2) | 0.73 | 0.80 |
| Choline  (mg/day) | 425* | 429  (340) | 345 (189) | 0.50 | 307  (327) | 319  (173) | 0.73 | 0.87 |
| Beta-carotene  (μg/day) | ND | 463  (2500) | 6210  (6672) | 0.07 | 3398  (9196) | 3346  (11035) | 0.36 | >0.99 |

Abbreviations: MDP, Mediterranean Diet Pattern; CHD, Canada’s Habitual Diet Pattern; IQR, interquartile range; RDA, Recommended Dietary Allowance; AI, Adequate Intake; MUFA, monounsaturated fatty acids; PUFA, polyunsaturated fatty acids; ND, no data. ^a^Wilcoxon-signed rank test, ^b^Mann-Whitney test, ^c^Niacin Equivalents (NE), ^d^Dietary Folate Equivalents (DFE), ^e^Retinol Activity Equivalents (RAE). Values represent median (IQR). Adequate intake (AI) are followed by an asterisk (*). Statistically significant difference p value <0.05.

Additional File 3. Fatty acid composition of the diets calculated as a percentage of total calories (females)

| *Females* | **MD Guidelines** | **MDP**  **Baseline** | **MDP**  **Week 12** | **CHD**  **Baseline** | **CHD**  **Week 12** |
| --- | --- | --- | --- | --- | --- |
| % Total Fat | 35 | 34 | 32 | 33 | 31 |
| % SFA | 13 | 12 | 10 | 13 | 10 |
| % MUFA | 15 | 15 | 14 | 14 | 13 |
| % n-6 PUFA | 6 | 6 | 7 | 6 | 7 |
| % n-3 PUFA | 1 | 1 | 1 | 1 | 1 |

Abbreviations: MDP, Mediterranean Diet Pattern; CHD, Canada’s Habitual Diet Pattern

Additional File 4. Median nutrient intakes of the male participants on the MDP and CHD at baseline and week 12

|  |  | **MDP**  **(n=6)** | | | **CHD**  **(n=4)** | | |  |
| --- | --- | --- | --- | --- | --- | --- | --- | --- |
| ***Male***  **(Median, IQR)** | **RDA/AI** | **Baseline** | **Week 12** | **P value within group^a^** | **Baseline** | **Week 12** | **P value within groups^a^** | **P value between intervention groups^b^** |
| Energy  (kcal/day) | 25-30 kcal/kg | 2865  (1971) | 3030 (3136) | >0.99 | 2249  (457) | 2267 (881) | 0.88 | 0.19 |
| Carbohydrates (g/day) | 130 | 294  (72) | 271  (252) | 0.81 | 275  (119) | 270  (98) | 0.25 | 0.90 |
| Protein  (g/day) | 56 | 99.6  (163.3) | 163  (241.7) | 0.44 | 88.9 (77.7) | 88.2 (46.8) | >0.99 | 0.11 |
| Total fat^f^  (g/day) | ND | 119  (121) | 154 (151.5) | 0.44 | 68.9 (21.2) | 89.5 (33.8) | 0.13 | 0.41 |
| Saturated fat (g/day) | ND | 29.6  (41.8) | 33.3  (43.9) | 0.63 | 24.3  (9.6) | 31  (7.3) | 0.38 | >0.99 |
| MUFA  (g/day) | ND | 37.2 (56.6) | 64  (72.2) | 0.63 | 22.7  (14) | 32.2  (15.8) | 0.13 | 0.29 |
| n-6 PUFA  (g/day) | 14-17 | 20.9  (16.3) | 26.2  (28.8) | 0.44 | 10.9  (10.8) | 16.2  (11.9) | 0.13 | 0.06 |
| n-3 PUFA  (g/day) | 1.6 | 3.96  (4.2) | 4.5  (5.2) | >0.99 | 1.43  (1.7) | 2.13  (2.0) | 0.13 | 0.73 |
| Total fibre  (g/day) | 30-38 | 24.4 (10.6) | 24.5 (17.4) | 0.81 | 25.8 (12.5) | 13.3 (10.5) | 0.13 | 0.11 |
| Sugars  (g/day) | < 25% energy | 132  (84.7) | 100  (60.7) | 0.63 | 120  (87) | 133  (35) | 0.88 | 0.41 |
| Sodium  (mg/day) | 1300-  1500* | 3470 (3735) | 3262  (5849) | 0.44 | 2940  (2418) | 2902 (1467) | 0.63 | 0.73 |
| Calcium  (mg/day) | 1000 | 814  (1226) | 1072 (334) | 0.63 | 1191 (1020) | 937  (657) | 0.13 | >0.99 |
| Iron  (mg/day) | 8 | 17  (7.3) | 20.3  (17.8) | >0.99 | 16.9  (7.4) | 11.8  (18.5) | 0.88 | 0.73 |
| Magnesium  (mg/day) | 400-  420 | 479  (318) | 697  (229) | 0.19 | 499  (290) | 315  (209) | 0.38 | 0.03 |
| Phosphorus  (mg/day) | 700 | 2311 (2737) | 2202  (1890) | 0.81 | 1844 (1267) | 1497  (711) | 0.38 | 0.06 |
| Zinc  (mg/day) | 11 | 13.3 (20.8) | 23.1  (34.8) | 0.44 | 12  (8.7) | 11.1  (8.3) | 0.63 | 0.42 |
| Copper  (mg/day) | 0.9 | 2.21  (1.1) | 3.3  (1.9) | 0.32 | 1.4  (0.9) | 1.3  (0.8) | 0.25 | 0.64 |
| Selenium  (μg/day) | 55 | 147  (229) | 292  (265) | 0.31 | 122  (40) | 123  (59.4) | >0.99 | 0.19 |
| Vitamin C  (mg/day) | 90 | 95  (140) | 144  (181) | 0.84 | 107  (100) | 97  (82) | >0.99 | 0.76 |
| Thiamin  (mg/day) | 1.2 | 2.2  (1.1) | 1.9  (0.8) | 0.57 | 2.7  (1.3) | 2.0  (1.1) | 0.63 | 0.76 |
| Riboflavin  (mg/day) | 1.3 | 2.7  (1.3) | 3.1  (1.2) | 0.69 | 2.5  (2.9) | 2.0  (0.9) | 0.38 | 0.04 |
| Niacin^c^  (mg/day) | 14 | 44  (49) | 35  (43) | >0.99 | 25  (6) | 22  (10) | 0.88 | 0.06 |
| Vitamin B6  (mg/day) | 1.3-  1.7 | 3.2  (3.9) | 3.0  (2.2) | >0.99 | 2.6  (1.1) | 2.0  (0.7) | 0.38 | 0.04 |
| Folate^d^  (DFE/day) | 400 | 541  (77) | 540  (156) | >0.99 | 472  (160) | 449  (192) | >0.99 | 0.17 |
| Vitamin B12  (μg/day) | 2.0 | 5.5  (12.9) | 6.6  (6.2) | >0.99 | 4.1  (6.9) | 4.0  (3.7) | 0.38 | 0.26 |
| Vitamin A^e^  (μg/day) | 900 | 799  (916) | 919  (391) | 0.44 | 1045 (579) | 703  (672) | 0.38 | 0.26 |
| Vitamin D  (μg/day) | 15 | 5.4  (15.6) | 9.9  (15.4) | 0.44 | 6.4  (3.5) | 5.5  (5.6) | 0.88 | 0.26 |
| Vitamin E  (mg/day) | 15 | 12  (14.3) | 18  (5.0) | 0.57 | 6.7  (7.5) | 7.7  (6.8) | 0.88 | 0.04 |
| Choline  (mg/day) | 550* | 499  (576) | 505  (276) | >0.99 | 348  (276) | 303  (130) | 0.63 | 0.04 |
| Beta-carotene  (μg/day) | ND | 864  (7414) | 5025  (5629) | 0.44 | 2771  (4777) | 1588  (1562) | 0.63 | 0.04 |

Abbreviations: MDP, Mediterranean Diet Pattern; CHD, Canada’s Habitual Diet Pattern; IQR, interquartile range; RDA, Recommended Dietary Allowance; AI, Adequate Intake; MUFA, monounsaturated fatty acids; PUFA, polyunsaturated fatty acids. ^a^Wilcoxon-signed rank test, ^b^Mann-Whitney test, ^c^Niacin Equivalents (NE), dietary Folate Equivalants (DFE), ^e^Retinol Activity Equivalents (RAE), ^f^Values appear are high as two participants were active males aged 18 and 22 years of age. Adequate intake (AI) are followed by an asterisk (*). Values represent median (IQR). Statistically significant difference p value <0.05.

Additional File 5. Fatty acid composition of the diets calculated as a percentage of total calories (males)

| *Males* | **MD Guidelines** | **MDP**  **Baseline** | **MDP**  **Week 12** | **CHD**  **Baseline** | **CHD**  **Week 12** |
| --- | --- | --- | --- | --- | --- |
| % Total Fat | 35 | 37 | 46 | 27 | 36 |
| % SFA | 13 | 9 | 10 | 10 | 12 |
| % MUFA | 15 | 12 | 19 | 9 | 13 |
| % n-6 PUFA | 6 | 7 | 8 | 4 | 6 |
| % n-3 PUFA | 1 | 1 | 1 | 1 | 1 |

Abbreviations: MDP, Mediterranean Diet Pattern; CHD, Canada’s Habitual Diet Pattern

**Additional File 6.** Fatty Acid Excretion

| **Fatty Acid**  (Males) | **MDP**  **(n=6)** | **CHD**  **(n=4)** | **Between Groups^a^** |
| --- | --- | --- | --- |
| SFA | 27 | 21 | 0.77 |
| MUFA | 43 | 47 | 0.77 |
| n-6 PUFA | 19 | 23 | 0.48 |
| n-3 PUFA | 0.47 | 1.7 | 0.51 |

| **Fatty Acid**  **(**Females) | **MDP**  **(n=9)** | **CHD**  **(n=9)** | **Between Groups^a^** |
| --- | --- | --- | --- |
| SFA | 27 | 22 | 0.55 |
| MUFA | 32 | 43 | 0.19 |
| n-6 PUFA | 21 | 16 | 0.67 |
| n-3 PUFA | 0.75 | 1.3 | 0.71 |

Abbreviations: MDP, Mediterranean Diet Pattern; CHD, Canada's Habitual Diet Pattern; IQR, interquartile range; SFA, saturated fatty acids; MUFA, monounsaturated fatty acids; PUFA, polyunsaturated fatty acids. ^a^Mann-Whitney test Statistically significant difference p value <0.05.

**Additional File 7.** CONSORT 2010 - Checklist

**Additional File 8.** The TIDierR Checklist
